# SARS-CoV-2 Fears Green: The Chlorophyll Catabolite Pheophorbide A Is a Potent Antiviral

**DOI:** 10.3390/ph14101048

**Published:** 2021-10-15

**Authors:** Guillermo H. Jimenez-Aleman, Victoria Castro, Addis Londaitsbehere, Marta Gutierrez-Rodríguez, Urtzi Garaigorta, Roberto Solano, Pablo Gastaminza

**Affiliations:** 1 National Centre for Biotechnology (CNB-CSIC), Department of Plant Molecular Genetics, 28049 Madrid, Spain; gh.jimenez@cnb.csic.es (G.H.J.-A.); addislonda@yahoo.es (A.L.); 2 National Centre for Biotechnology (CNB-CSIC), Department of Cell & Molecular Biology, 28049 Madrid, Spain; vcastro@cnb.csic.es (V.C.); ugaraigorta@cnb.csic.es (U.G.); 3 Medicinal Chemistry Institute (IQM-CSIC), Department of Biomimetics for Drug Discovery, 28006 Madrid, Spain; mgutierrez@iqm.csic.es

**Keywords:** Pheophorbide a, COVID-19, SARS-CoV-2, chlorophyll, liverwort, *Marchantia polymorpha*

## Abstract

SARS-CoV-2 pandemic is having devastating consequences worldwide. Although vaccination advances at good pace, effectiveness against emerging variants is unpredictable. The virus has displayed a remarkable resistance to treatments and no drugs have been proved fully effective against COVID-19. Thus, despite the international efforts, there is still an urgent need for new potent and safe antivirals against SARS-CoV-2. Here, we exploited the enormous potential of plant metabolism using the bryophyte *Marchantia polymorpha* L. and identified a potent SARS-CoV-2 antiviral, following a bioactivity-guided fractionation and mass-spectrometry approach. We found that the chlorophyll derivative Pheophorbide a (PheoA), a porphyrin compound similar to animal Protoporphyrin IX, has an extraordinary antiviral activity against SARS-CoV-2, preventing infection of cultured monkey and human cells, without noticeable cytotoxicity. We also show that PheoA targets the viral particle, interfering with its infectivity in a dose- and time-dependent manner. Besides SARS-CoV-2, PheoA also displayed a broad-spectrum antiviral activity against enveloped RNA viral pathogens such as HCV, West Nile, and other coronaviruses. Our results indicate that PheoA displays a remarkable potency and a satisfactory therapeutic index, which together with its previous use in photoactivable cancer therapy in humans, suggest that it may be considered as a potential candidate for antiviral therapy against SARS-CoV-2.

## 1. Introduction

The pandemic caused by the severe acute respiratory syndrome coronavirus 2 (SARS-CoV-2) is having devastating consequences, with more than 229M infected people and over 4.7M deaths worldwide (https://covid19.who.int/, accessed on 25 September 2021). Besides the humanitarian cost, this pandemic carries a tremendous negative economic impact, a huge challenge for any government to overcome. The coronavirus disease 2019 (COVID-19), the respiratory illness caused by SARS-CoV-2 (Genus *Betacoronavirus*; Subgenus *Sarbecovirus*), has displayed a remarkable resistance to treatments and no drugs have been proved fully effective against the virus. Moreover, the COVID-19 pandemic has made evident the need for a global strategy to fight similar situations that may appear in the future.

Current efforts to eradicate COVID-19 are focused on the development of vaccines and the search for antiviral lead compounds, mainly repurposing of existing drugs. Although the vaccination campaign seems to advance at good pace, its effectiveness against some of the present and future strains of SARS-CoV-2 is hard to predict due to the existence of different strains that could drastically reduce the vaccine efficiency [1]. In addition, the best anti-COVID-19 drugs approved so far (e.g., Remdesivir (RMDV), Favipiravir or its derivative Avifavir), have shown only a mild effect against the virus, slightly reducing hospitalization time of patients [2]. Other treatments, such as Chloroquine and Hydroxychloroquine, appear to help at least a subgroup of patients, but the possible negative side effects of these drugs remain under investigation [3]. Although, some compounds (e.g., Aplidin, Mefloquine, Nelfinavir, Protoporphyrin IX and Verteporfin) have shown potential on in vitro assays, and some of them also in animal models [4,5,6], there is an urgent need for new potent and safe antivirals against SARS-CoV-2. Noteworthy, new pathogens, including viruses, are expected to emerge in coming decades, which puts an enormous pressure on society in order to be ready to fight back future pandemics with the proper chemical, biological, and engineering tools, including effective new antivirals.

For centuries, medical needs of society have been widely covered by plants, which have an extremely rich metabolism that provides them with a wide repertoire of chemical weapons to cope with environmental biotic stresses, including viruses [7,8]. Originally recognized by traditional medicine, plants are the main source of compounds used today in pharmacology, from salicylates (from Salix sp.) to current anticancer drugs (e.g., Vinblastine and Vincristine from *Vinca* sp., or Taxol and Paclitaxel from *Taxus baccata.* L), simply to cite a few successful examples [9,10]. Therefore, the identification of new plant sources of enzymatic variants and metabolites is essential to the discovery of new drugs and their optimization by metabolic engineering. Aromatic and exotic vascular plants are commonly studied in order to identify pharmacologically interesting compounds. In contrast, the metabolic richness of bryophytes (non-vascular plants including mosses, liverworts and hornworts) has been little explored. Bryophytes are rarely attacked by pathogens (fungi, bacteria, viruses) or herbivores (insects, snails, mammals) in their natural habitats, which indicates that they are well protected by a potent arsenal of secondary defense metabolites. However, studies on their chemical constituents have been neglected until recently [11]. Indeed, only around 5% of bryophyte species have been metabolically explored, and results have shown an enormously rich diversity of secondary metabolites, particularly in liverworts [12]. Strikingly, more than 1600 terpenoids have been reported in liverworts, whereas only about 100 terpenoids have been identified in the medicinal plant *Cannabis sativa* L. [11,12,13]. More importantly, several liverwort species of the order Marchantiales, including *Marchantia polymorpha* L., produce terpenoids and bisbibenzyls with enormous potential for pharmaceutical applications since they show remarkable antimicrobial, antioxidant, cytotoxic, anticancer and antiviral (anti-HIV) activities [11,12,13,14,15,16]. Therefore, we made use of our vast experience in virology and *Marchantia*’s hormonal signaling and secondary metabolism in order to explore this plant’s potential as a source of antiviral metabolites, particularly, against the SARS-CoV-2 virus.

In this study, we employed a set of *Marchantia* wild type plants, and signaling and metabolic mutants to systematically study the pharmacological potential of this liverwort. We found that total extracts from all the plants displayed a remarkable antiviral activity against SARS-CoV-2. Using a bioactivity-guided chromatographic approach, in addition to mass-spectrometry (MS), we identified the antiviral metabolite as Pheophorbide a (PheoA), a porphyrin chlorophyll derivative very similar to animal Protoporphyrin IX, also described as a strong antiviral [6]. We also found that PheoA has a broad-range antiviral activity against enveloped RNA viruses and acts as a virucidal by directly acting on the viral particle. Furthermore, PheoA’s antiviral activity is additive to that of RMDV; this places PheoA (with very low toxicity) as a suitable candidate for antiviral therapy against SARS-CoV-2.

## 2. Results

### 2.1. Crude Extracts of M. polymorpha Show Anti-SARS-CoV-2 Activity

**Figure 1:**
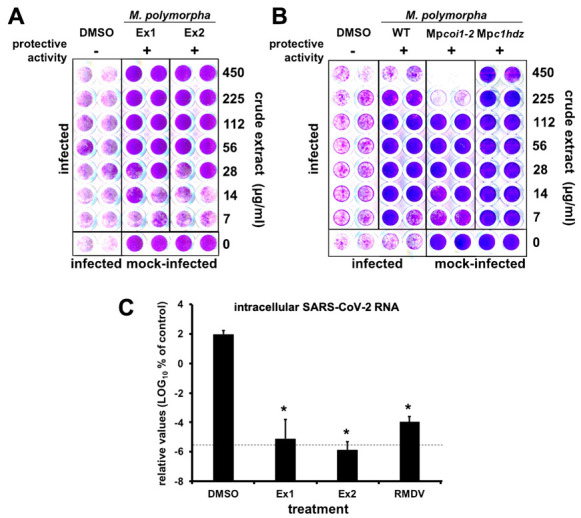
*M. polymorpha* extracts interfere with SARS-CoV-2-induced cytopathic effect and virus propagation. (**A**,**B**) Vero E6 cells were inoculated with SARS-CoV-2 (MOI = 0.001) in the presence of serial 2-fold dilutions of crude extracts from (**A**) two different *M. polymorpha* ecotypes, *ruderalis* (Ex1) and *polymorpha* (Ex2) or (**B**) WT, *Mpcoi1-2* or *Mpc1hdz Marchantia* plants, incubated for 72 h, time after which they were fixed and stained with a crystal violet solution. Mock-infected cells were used as the control of the integrity of the cell monolayer. Images show a representative experimental plate comparing *M. polymorpha* extracts with vehicle (DMSO)-treated cells. (**C**) Vero E6 cells were inoculated with SARS-CoV-2 (MOI = 0.001) in the presence of vehicle (DMSO), RMDV (25 µM) or a 100 µg/mL dilution of crude extracts. Uninfected samples were used as the control (mock). Total RNA was extracted 72 h post-infection and subjected to RT-qPCR to determine viral load. The dotted line indicates the limit of detection of the assay. Normalized viral RNA levels are shown as percentage of the viral RNA found in vehicle-treated cells. Data are shown as mean (± SD) of three biological replicates. Statistical significance was estimated using one-way ANOVA and a Dunnet’s post hoc test (* *p* < 0.05).

In order to explore for the presence of anti-SARS-CoV-2 metabolites in *M. polymorpha*, we prepared crude extracts of two different *M. polymorpha* subspecies (subsp. *ruderalis* from Japan and subsp. *polymorpha*, from Spain). The anti-SARS-CoV-2 activity of the resulting crude extracts was tested in Vero E6 cell monolayers infected with the SARS-CoV-2 NL2020 strain (Figure 1A). Infection was carried out at a multiplicity of infection (MOI) of 0.001 for 72 h. In the absence of antiviral activity, SARS-CoV-2 infection triggers cell death of Vero E6 cells and results in loss of cell biomass in the well, which is readily visualized as a strong reduction in crystal violet staining (DMSO, Figure 1A). Treatment of the cells during infection with RMDV (positive control), the only clinically approved antiviral for treatment of COVID-19 patients, protected the cell monolayers down to its reported EC_50_ of 1.5 μM (Supplementary Figure S1). Thus, protective activity may be used as a rapid qualitative surrogate assay to facilitate identification of substances with antiviral potential [17].

Treatment of cell cultures with a broad range of concentrations of *Marchantia* crude extracts resulted in cell protection against virus-induced cytopathic effect without any signs of cytotoxicity at the assayed concentrations (Figure 1A, Ex1 and Ex2), suggesting the presence of one or more *Marchantia* metabolites with strong antiviral activity.

### 2.2. Marchantia Extract Bioactivity Depends on Plant’s Primary Metabolism

Before isolation of active compound(s) and quantitation of antiviral activity of the bioactive metabolite(s), we explored whether the putative antiviral metabolite(s) could belong to plant’s secondary metabolism. Jasmonates (JAs) are a family of oxylipin-derived phytohormones regulating many aspects of plant development and growth; as well as mediating defense responses through transcriptional activation of the secondary metabolism, which includes several classes of compounds such as alkaloids, terpenoids and flavonoids [18,19,20]. In *Marchantia*, secondary metabolites accumulate in specific organelles named oil bodies (OB), which are confined to scattered idioblastic OB cells distributed throughout the thallus [21]. Therefore, we tested extracts from *M. polymorpha* WT, Mp*coi1-2* [impaired in dn-OPDA perception, the active jasmonate in *Marchantia* [19], thus, in defense metabolite induction], and Mp*c1hdz* plants (impaired in OB formation; MpC1HDZ is a transcription factor required for OB cells differentiation). Mp*c1hdz* mutants render plants defective in secondary metabolites, thus, susceptible to herbivory and microbes [22]. Although some differential cytotoxicity was observed in the different plants at high extract concentrations, likely due to the presence of different metabolites in the extracts, all tested *Marchantia* extracts showed clear protective activity below cytotoxic doses (Figure 1B), suggesting that the active antiviral is present in all three plants and should not belong to the plants’s secondary metabolism. Indeed, data in Figure 1B suggests that the activity of extracts is due to the presence of a metabolite (or metabolites) that is constitutively synthesized and/or derived from the plant’s primary metabolism. Remarkably, regulation of primary metabolism is achieved in all plants by very similar conserved metabolic pathways [23,24]. To strengthen this hypothesis, we tested crude extracts of several plant species (sweet amber (*Hypericum androsaemum* L.), fern (*Blechnum spicant* (L.) Sm.), nettle (*Urtica dioica* L.), moss (*Physcomitrium patens* (Hedw.) Mitt.), tobacco (*Nicotiana benthamiana* Domin) and thale cress (*Arabidopsis thaliana* (L.) Heynh.) for their capability of providing protection to Vero E6 cells against the SARS-CoV-2 virus and showed clear protection in most cases (Supplementary Materials Figure S2), further supporting the notion that the bioactive molecule(s) correspond to plant primary metabolism (Figure 1B).

Given that potential antiviral activity had been identified by an indirect, qualitative readout, we set out to define if *Marchantia* extracts were indeed capable of interfering with viral spread in cell culture by determining viral load after virus spread in a multiple cycle infection experiment. Thus, Vero E6 cells were inoculated at MOI of 0.001 in the presence of control-solvent, RMDV (25 µM) and *Marchantia* extracts from Figure 1A (~100 µg/mL). Viral RNA load, which in this experimental setup represents the degree of virus propagation, was determined 72 h post infection by RT-qPCR (Figure 1C). In the vehicle control (DMSO), viral RNA accumulated six orders of magnitude above the assay limit of detection (Figure 1C), whereas the viral RNA was undetectable in samples treated with the antiviral RMDV. Importantly, in samples treated with *Marchantia* extracts, the viral RNA levels were comparable to those observed upon RMDV treatment, that is, close to the assay’s detection limit (Figure 1C), suggesting the presence of at least one antiviral compound that strongly prevented SARS-CoV-2 infection and/or spread and, thus, reduced virus-induced CPE (Figure 1).

### 2.3. Identification of the Antiviral Metabolite

**Figure 2:**
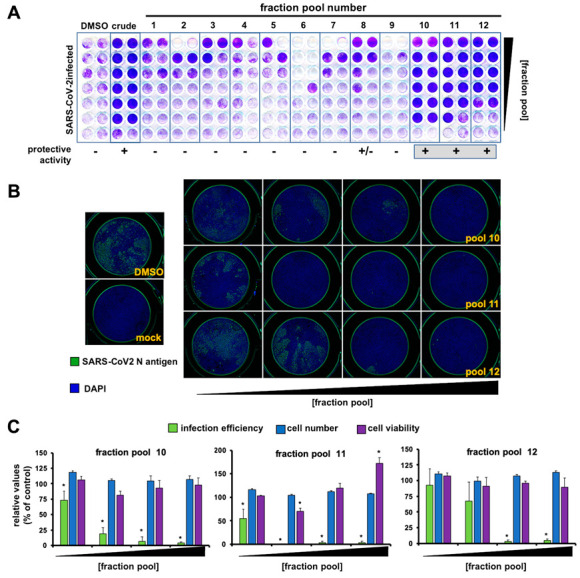
Extract fractionation and identification of antiviral fraction pools. (**A**) Vero E6 cells were inoculated with SARS-CoV-2 (MOI = 0.001) in the presence of serial 2-fold dilutions of vehicle (DMSO), a crude *Marchantia* extract and the fraction pools. Inoculated cultures were incubated for 72 h, time after which they were fixed and stained with a crystal violet solution. Mock-treated cells were used as the control of the integrity of the cell monolayer (non-infected). (**B**) Vero E6 cells were inoculated (MOI = 0.01) in the presence of serial dilution of the fractions and incubated for 24 h before fixation; processing for immunofluorescence microscopy and cytotoxicity assays was as described in the materials and methods section. Representative images of the fraction pool cell-based evaluation are shown. (**C**) Quantitation of infection efficiency, cell number and cell viability. These data are shown as average (± SD) of three biological replicates. Statistical significance was estimated using one-way ANOVA (Dunnet´s post hoc test, * *p* < 0.05).

In order to identify the bioactive metabolite(s), we followed a bioactivity-guided chromatographic fractionation of the *Marchantia*’s WT crude extracts, which showed clear antiviral potential in previous assays. Chromatographic fractions were obtained via flash column employing a solvent polarity gradient, starting at *n*-hexane (100%) up to AcOEt:MeOH (4:1, *v*/*v*). A total of 56 fractions were obtained; fractions of a similar composition, based on their thin layer chromatography (TLC) profiles were combined and evaluated as 12 new pooled fractions (from now on, Fractions 1 to 12, Figure 2A) to identify the fractions displaying antiviral activity. Most fractions did not show any clear protection and Fraction 6 was particularly cytotoxic (Figure 2A). Fractions 10, 11 and 12 showed clear protective activity in the monolayer protection assay (Figure 2A), suggesting that they contain antiviral compounds. To directly confirm their antiviral activity, the viral antigen load reduction after inoculation of cell cultures with SARS-CoV-2 (MOI = 0.01) was measured. In this experimental setup, viral antigen accumulates as a consequence of virus propagation and can be quantitated using automated immunofluorescence microscopy. Figure 2B shows a dose-dependent reduction in viral antigen accumulation and the absence of cytotoxicity, as confirmed by normal cell numbers, estimated by DAPI staining and image analysis (Figure 2C), and cell viability studies performed in parallel in uninfected cultures by MTT assays (Figure 2B,C).

Interestingly, TLCs of the three active fractions presented red fluorescent spots (under long-wave ultraviolet light, 365 nm; Supplementary Materials Figure S3A), which are characteristic of plant chlorophylls, but with a smaller retention factor (Rf = 0.36, AcOEt:MeOH (9:1, *v*/*v*)) than that of chlorophyll (Rf = 0.92). At this point, we suspected that the active antiviral metabolite(s) could be related to plant chlorophylls, because of (i) relative antiviral potency coincides with the relative abundance of the red fluorescent spots (Figure 2C and Supplementary Materials Figure S3) and (ii) a very weak protective activity was observed at low dilutions of fractions pools 2 and 3 (Figure 2A and Supplementary Materials Figure S3B), both containing chlorophyll. Indeed, these fractions, when re-chromatographed, yielded spots on the TLC with a Rf consistent with that observed in the active fractions 10 to 12, indicating that the active metabolite is a chlorophyll derivative (Supplementary Materials Figure S3B). It is worth mentioning that heat notably accelerated the chlorophyll decomposition into the investigated metabolite (Supplementary Materials Figure S3B).

**Figure 3:**
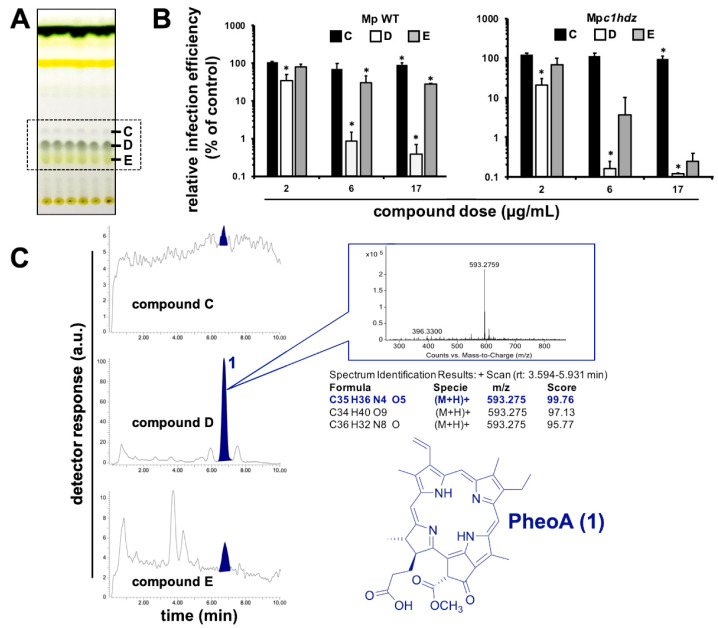
Identification of the main antiviral compound in *Marchantia* extracts. (**A**) Representative TLC of *Marchantia* WT and *c1hdz* extracts. Compounds C, D and E (box) were tested for their antiviral potential. (**B**) Vero E6 cells were inoculated (MOI = 0.01) in the presence of the indicated compounds and incubated for 24 h before fixation and processing for immunofluorescence microscopy. Data are shown as average (± SD) of three biological replicates. Statistical significance was estimated using one-way ANOVA and a Dunnet´s post hoc test (* *p* < 0.05). (**C**) Representative HPLC/MS analysis (shown for WT) of fractions C, D, and E, including exact mass determination of the antiviral candidate 1, and its inferred chemical structure.

Next, we employed preparative TLC in order to better isolate and characterize the red-light emitting metabolite. Photosynthetic metabolites were extracted from *M. polymorpha* WT and Mp*c1hdz* plants, and the extracts subjected to preparative TLC, to further verify that the investigated molecule is indeed a primary metabolite present both in WT and mutant *Marchantia* plants. Three spots showed fluorescence in the vicinity of the expected Rf; these spots where isolated, analyzed by HPLC-UV-MS and the bioactivity assayed as fractions C, D and E (Figure 3A). Similar spot concentrations were studied using a quantitative method in order to identify the molecule(s) with the strongest antiviral potential. Fraction D, which corresponded with an enrichment of compound **1** in the UV chromatograms (Figure 3C), showed the strongest antiviral activity, as compared with C or E (Figure 3B). Careful analysis of the MS spectra revealed a molecular formula of C_35_H_36_N_4_O_5_ for compound **1**, as deduced by HR-ESI^+^-MS from its monoprotonated molecular ion, [M + H]^+^, with a *m*/*z* of 593.2759. The identified molecular formula (containing four nitrogen atoms), the exact mass and the characteristic red fluorescence of the compound helped to identify 1 as the chlorophyll catabolite PheoA (Figure 3C). The identity of PheoA was further confirmed by comparison with both a commercially available original standard (Santa Cruz Biotechnology, Dallas, TX, USA) and a semisynthetic sample. PheoA was obtained semi-synthetically from *Marchantia* in good overall yield via a solvent free, thus, environmentally friendly method (Supplementary Materials Figure S4 and M&M section).

### 2.4. Antiviral Activity of PheoA

To confirm the antiviral potential of PheoA, a commercially available PheoA stock solution was serially diluted and mixed with a virus stock to inoculate Vero E6 and Huh7-ACE2 cells (human hepatoma cells expressing ACE2). Cells were fixed 72 h post-inoculation and stained with crystal violet to visualize the integrity of the cell monolayer. Figure S5 shows consistent protective capacity of PheoA above 65 nM in both cell lines (Supplementary Materials Figure S5).

**Figure 4:**
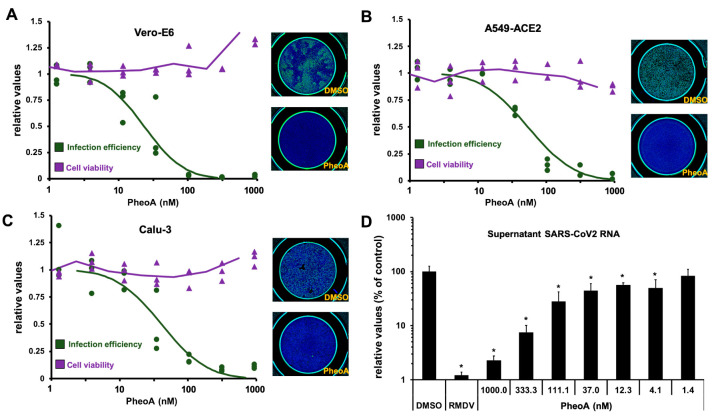
PheoA shows antiviral activity against SARS-CoV-2 in Vero E6 cells and human lung epithelial A549-ACE2 and Calu3 cell lines. (**A**–**C**) Commercially available PheoA was serially diluted and mixed (1:1, *v*/*v*) with SARS-CoV-2 preparations to achieve the indicated compound concentrations and a final MOI of 0.005 for (**A**) Vero E6 and (**B**) Calu3 and 0.01 for (**C**) A549-ACE2 cells. Cultures were incubated for 48 h, fixed and processed for automated immunofluorescence microscopy analysis. Parallel, uninfected cultures were processed for cytotoxicity evaluation using an MTT assay. Relative infection efficiency data (*n* = 3 per dose) are shown as individual data and a PROBIT regression curve (green line) using the represented values. Cytotoxicity data (*n* = 3 per dose) are shown as the individual data and a moving average trend line. (**D**) A549-ACE2 cells were inoculated at MOI = 0.01 in the presence of increasing concentrations of PheoA or RMDV (5 µM) and incubated for 48 h. Samples of the supernatants were collected, heat-inactivated and directly subjected to RT-qPCR to estimate overall infection efficiency. Data are expressed as relative values compared with the vehicle (DMSO)-treated cells and are shown as the mean (± SD) of three biological replicates (*n* = 3). Statistical significance was estimated using one-way ANOVA and a Dunnet´s post hoc test (* *p* < 0.05).

PheoA antiviral activity was further confirmed by immunofluorescence microscopy–to estimate virus propagation–and an MTT assay–to evaluate compound cytotoxicity. PheoA dose–response curves demonstrate PheoA’s antiviral activity against SARS-CoV-2 in Vero E6 and human lung epithelial cells (A549-ACE2 and Calu3); in all three models, no cytotoxicity was observed at the assayed doses (Figure 4A–C). This dataset was used to determine effective concentrations (EC_50_ and EC_90_) and cytotoxicity indexes. The estimated EC_50_ and EC_90_ values were around 50 nM and 100 nM, respectively, with a wide therapeutic window in all tested cell lines (Table 1)

To independently evaluate the extent to which PheoA interferes with SARS-CoV-2 infection (i.e., with viral replication), we employed RT-qPCR to determine the extracellular viral RNA load in human cells inoculated with SARS-CoV-2 (MOI = 0.001). In this experimental setup, RMDV (5 µM) reduced extracellular viral RNA by two orders of magnitude. Interestingly, PheoA reduced viral RNA accumulation in a dose-dependent manner after a 48 h incubation period (Figure 4D). These results indicate that PheoA displays a remarkable potency and a satisfactory therapeutic index and suggest that it may be considered as a potential candidate for antiviral therapy against SARS-CoV-2. Furthermore, results suggest that PheoA is a major determinant of the antiviral activity observed in crude *Marchantia* extracts (Figure 1). This notion is underscored by the fact that the semisynthetic PheoA (ca. 90% by HPLC-UV/Vis) showed comparable potency to crude extracts in the different cell lines (Supplementary Materials Figure S6). Nevertheless, other related chlorophyll metabolites may also contribute with antiviral activity. In fact, pyropheophorbide a (pPheoA), which was also found in antiviral fractions, was tested to verify its antiviral potential in Vero E6 cells. The pPheoA showed antiviral activity in the absence of measurable cytotoxicity with an EC_50_ of 185 nM (Supplementary Materials Figure S7), suggesting that pPheoA may contribute to the overall antiviral activity of the extracts.

### 2.5. Antiviral Spectrum of PheoA

**Figure 5:**
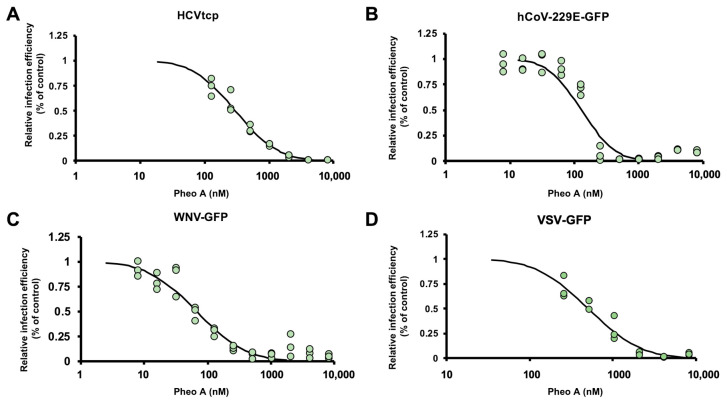
Antiviral spectrum of PheoA against different RNA viruses. The effectiveness of PheoA was tested against four different recombinant RNA viruses expressing reporter genes. (**A**) Huh7 cells were infected with HCVtcp in the presence of increasing PheoA doses and luciferase activity was determined 48 h post-inoculation. (**B**–**D**) Cells were inoculated in the presence of increasing concentrations of PheoA at MOI 0.01 and incubated to enable virus propagation. At the endpoint, cells were fixed and counter stained with DAPI to control for unexpected cytotoxic effects. Relative infection efficiency was estimated using automated microscopy and is expressed as percentage of the infection efficiency observed in control wells. (**B**) Huh7 cells were infected with hCoV-229E-GFP and fixed 48 h post-inoculation. (**C**) Huh7 cells were infected with WNV-GFP and fixed 48 h post-inoculation. (**D**) A549-ACE2 cells were inoculated with VSV-GFP and fixed 16 h post-inoculation. Individual replicate data are shown as green dots (*n* = 3) and the PROBIT regression curve used to estimate EC50 values is shown.

PheoA has previously been proven as an antiviral against the hepatitis C virus (HCV) [25] and virucidal against herpes simplex virus (HSV) [26]. Thus, we determined the antiviral spectrum of PheoA on different enveloped RNA viruses. First, we confirmed antiviral activity against HCV, using a surrogate model of infection by propagation-deficient, bona fide reporter virus bearing a luciferase reporter gene generated by trans-complementation (HCVtcp). Dose–response curves of the luciferase activity in HCVtcp-infected Huh7 cells indicated an EC_50_ of 300 nM for PheoA against HCV (Figure 5A), very similar to the previously reported EC_50_ [25].

Next, we asked whether PheoA antiviral activity against SARS-CoV-2 could also be observed against other human coronaviruses such as hCoV-229E (Genus *Alphacoronavirus*; Subgenus *Duvinecovirus*), which has been associated with mild respiratory infections [27]. Huh7 cells were inoculated with a GFP reporter-expressing recombinant hCoV-229E (MOI = 0.01) and total GFP expression in the target Huh7 cells was evaluated by automated microscopy 48 h post-inoculation. Similar to the results with the HCV infection model, PheoA reduced viral spread (EC_50_ of 128 nM) while no cytotoxicity was observed (Figure 5B). Comparable results were obtained in an experimental model of infection by the West Nile Virus, a mosquito-borne zoonotic Flavivirus that may cause encephalitis in infected humans. A recombinant, di-cistronic infectious molecular clone expressing GFP in the second cistron [28] was used to inoculate Huh7 cells (MOI = 0.01). Cells were imaged 48 h post infection and the degree of virus propagation was determined via automated microscopy; dose response curves (Figure 5C) indicated antiviral activity for PheoA with an EC_50_ of 64 nM). Finally, PheoA antiviral activity was observed against a recombinant vesicular stomatitis virus bearing a GFP reporter gene (VSV-GFP) obtaining comparable effective concentrations in Huh7 (EC_50_ 506 nM; Figure 5D) and A549-ACE2 cells (EC_50_ 578 nM; data not shown), further reinforcing the notion that PheoA is a broad-spectrum antiviral molecule with activity against all tested viruses, including SARS-CoV-2.

### 2.6. PheoA Can Be Employed in Combination with RMDV

**Figure 6:**
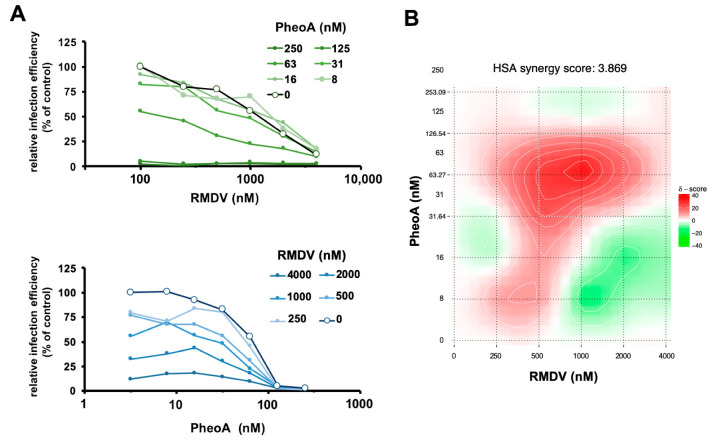
Combination treatment of PheoA with RMDV. Vero E6 cells were inoculated at MOI = 0.005 in the presence of increasing concentrations of PheoA in combination with increasing doses of RMDV. Twenty-four hours post infection, cells were fixed and processed for automated immunofluorescence microscopy. Relative infection efficiency values were estimated as percentage of the values obtained in mock-treated cells. (**A**) Data are shown as average of two biological replicates. (**B**) Heatmap describing the areas of synergy within the combination treatments.

Once the broad antiviral activity of PheoA has been demonstrated, we studied whether the addition of PheoA to RMDV treatment could result in a synergistic effect on viral infection. Thus, combination treatments were performed with increasing doses of PheoA and RMDV. Drugs were mixed in different proportions, combined with infectious SARS-CoV-2 (MOI = 0.01) and the mixtures were used to inoculate Vero E6 cells. Twenty-four hours later, cells were fixed and processed to determine the infection efficiency as using immunofluorescence microscopy. Individual treatment with either compound resulted in the expected dose-dependent inhibition of virus infection, achieving the EC_50_ at the expected doses (2000 nM for RMDV and 40 nM for PheoA). Increasing concentrations of PheoA improved RMDV efficacy and viceversa. However, full analysis of the combinations resulted in a synergy index close to three, indicating that the drug combination is mostly additive [29], with an area of synergy at concentrations close to the EC_50_ (Figure 6). These results suggest that combinations of PheoA with other antivirals may result beneficial, as it was observed by its additive effect in combination with RMDV in cell culture infection models.

### 2.7. Characterization of PheoA Mode of Action on SARS-CoV-2 Infection

**Figure 7:**
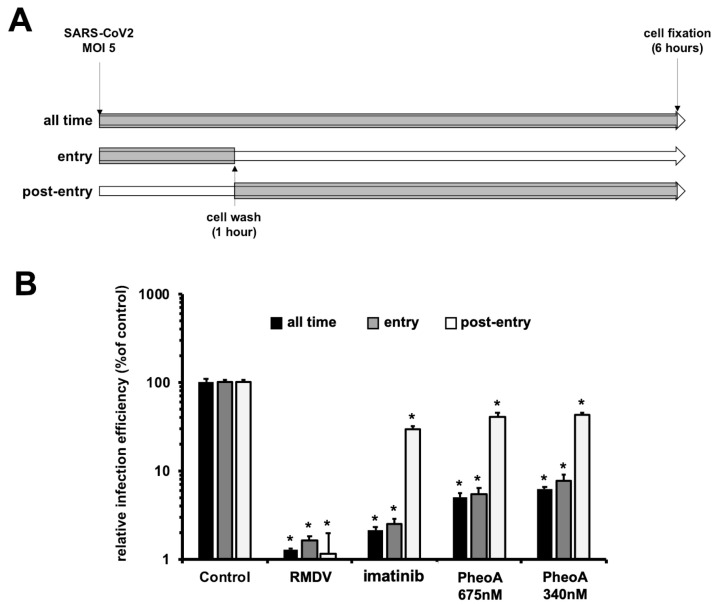
Time-of-addition experiments indicate that PheoA interferes with early aspects of SARS-CoV-2 infection. Vero E6 cells were inoculated at MOI = 5 in the presence (gray) or absence (white) of the indicated doses of PheoA, RMDV or imatinib as described in both the text and the scheme. Cells were incubated for 6 h in the presence (gray) or absence (white) before chemical fixation and processing for immunofluorescence microscopy. (**A**) Schematic diagram of the times where compound was present in the assay. (**B**) Infection efficiency is expressed as the percentage of that observed in vehicle DMSO-treated cells and is shown as average and standard deviation of three biological replicates (*n* = 3). Statistical significance was estimated using one-way ANOVA and a Dunnet´s post hoc test (* *p* < 0.05).

Next, antiviral efficacy of PheoA was compared when PheoA was (i) present at all times, (ii) added only during virus inoculation, or (iii) added only after virions had effectively penetrated the cells in single-cycle infection experiments (MOI = 5). Imatinib (15 µM), for which antiviral activity at the level of virus entry has previously been demonstrated [17] and RMDV were employed as the reference compounds. Infection efficiency revealed the expected antiviral activity for imatinib, RMDV and PheoA when maintained at all times in the experiment (Figure 7). RMDV was highly effective at reducing infection efficiency, as suggested by SARS-CoV-2 N protein staining, indicating that the measured signal requires viral RNA replication (Figure 7B). Imatinib showed comparable efficacy when added during the virus entry phase and greatly lost efficacy when added after virion internalization, as expected for an entry inhibitor [17]. Similar results were obtained with PheoA, inhibition was nearly identical when maintained at all times or added during viral entry, but only ca. 6% of the maximum efficacy was observed when added after virion internalization. These results suggest that PheoA is mainly acting at early stages of the infection, potentially at the level of viral entry.

In view of these results, we directly tested this hypothesis by determining the antiviral activity of PheoA in a surrogate model of infection recapitulating only aspects related with viral entry such as receptor recognition, virion internalization or membrane fusion. This system is based on reporter retroviral vectors pseudotyped with SARS-CoV-2 Spike protein (SARS2pp) or VSV glycoprotein as a control (VSVpp). Infection efficiency in the presence of antiviral molecules is determined as the relative expression values of the reporter gene, in this case a Firefly luciferase [17]. Relative infection efficiency was measured in the presence of the entry inhibitor imatinib (15 µM) and antiviral doses of PheoA. As expected, imatinib selectively inhibited SARS2pp and not VSVpp entry (Supplementary Materials Figure S8). PheoA barely interfered with either of the retroviral pseudotype infection efficiencies, with a maximum reduction of 40% at the maximum dose (675 nM) (Supplementary Materials Figure S8). These results suggest that, while time-of-addition experiments suggest that PheoA interferes predominantly with early aspects of the infection, surrogate models of viral entry indicate that it does not interfere substantially with molecular events leading to viral entry per se.

**Figure 8:**
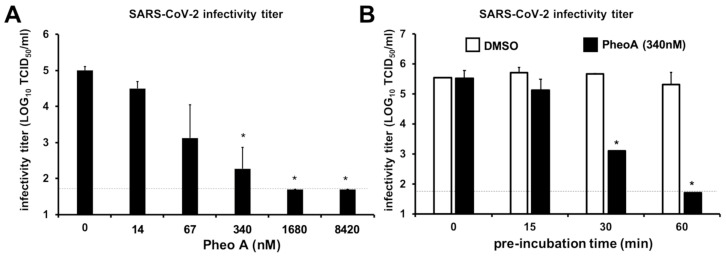
PheoA interferes with SARS-CoV-2 virion infectivity in a dose- and time-dependent manner. SARS-CoV-2 virus stocks were diluted to obtain 1 × 10^5^ TCID_50_/mL and were mixed with increasing concentrations of PheoA or the vehicle (DMSO). (**A**) Dose-dependent reduction in SARS-CoV-2 infectivity by PheoA. Virus-compound mixtures were incubated at room temperature for 30 min and were serially diluted to determine the remaining infectivity titer using endpoint dilution and determination of virus-induced cytopathic effect by crystal violet staining in Vero-E6 cells. (**B**) Pre-incubation time-dependent reduction in SARS-CoV-2 infectivity by PheoA. Experiments were carried out using a fixed dose of PheoA (340 nM) and increasing pre-incubation times before serial dilution for TCID_50_ determination. Values are expressed as LOG TCID_50_/mL and shown as the average and standard deviation of three independent experiments (*n* = 3). Statistical significance was estimated using one-way ANOVA and a Dunnet’s post hoc test (* *p* < 0.05).

PheoA irreversibly interferes with the virion infectivity in HSV and influenza infection models [30], therefore, we explored whether PheoA could be virucidal for SARS-CoV-2 virions, a property that would be compatible with the observations made in the time-of-addition experiments (Figure 7). Thus, a known number of infectious particles (10^5^ TCID_50_/mL) were mixed with increasing doses of PheoA [14 to 8420 nM)] and incubated for 30 min before residual infectivity titer was calculated by endpoint dilution and TCID_50_ determination. The dose of PheoA was kept below its effective concentrations during the titration experiment. Figure 8A shows how pre-incubation of the infectious virions with PheoA resulted in a dose-dependent reduction in the remaining virus infectivity that was statistically significant at doses above 67 nM. Next, we tested the impact of incubation time at a fixed PheoA dose (340 nM). Incubation of the virus at room temperature for up to 15 min in the presence of PheoA did not have any significant impact on virus infectivity (Figure 8B). However, longer incubation periods reduced partially (30 min) or below detection limits (60 min) SARS-CoV-2 infectivity. The fact that exposure to PheoA for less than 30 min does not reduce SARS-CoV-2 infectivity, testifies for the lack of interference of the diluted PheoA on viral infection efficiency. Thus, these results reveal a time- and dose-dependent reduction in virus infectivity by exposure to PheoA, suggesting that PheoA is virucidal for SARS-CoV-2 virions and that virion infectivity inactivation contributes to its overall antiviral effect.

## 3. Discussion

Due to their metabolic richness plants have been traditionally used as source of medicines. The potential of plant metabolites in pharmacology is still far from being saturated, particularly in certain plant clades. Indeed, bryophytes (non-vascular plants) are particularly rich in specialized metabolites that are rarely found in other plant lineages [26]. Here, we explored this richness in order to find antiviral compounds against the SARS-CoV-2 virus by employing an activity-guided chromatographic method; and identified PheoA as a potent antiviral, very efficient not only against SARS-CoV-2 but also against several other enveloped viruses.

The first evidence for antiviral activity of PheoA derives from observations made on HSV infection models [27]. In those initial reports, some degree of selectivity towards other viruses was reported, since adenovirus (Type VI), Japanese Encephalitis virus (JEV) or poliovirus were not affected by treatment with PheoA-enriched algal extracts [31]. Subsequent studies suggested that PheoA and pPheoA display broad-spectrum antiviral activity against enveloped viruses, including influenza A [30] and HIV [28], but not against non-enveloped viruses [30]. Ohta et al., reported that PheoA-containing preparations may display virucidal activity against HSV [32], a concept that was further supported by Bouslanu et al. [30]. Our observations support that PheoA inactivates SARS-CoV-2 virion infectivity through a virucidal mode of action. First, time-of-addition experiments indicate that early aspects of the infection are targeted by PheoA. Second, the study of viral entry using retroviral pseudotypes did not reveal any measurable antiviral activity against SARS-CoV-2 entry, suggesting that receptor recognition by the Spike protein, particle internalization and Spike-mediated fusion are not overtly affected by PheoA. Similar models have been used to identify key SARS-CoV-2 entry factors as well as to study antibody neutralizing activity [33,34]. Thus, inactivation of viral infectivity (virucidal activity) was tested as a possible mechanism reconciling these apparently contradicting observations. Pre-incubation of infectious SARS-CoV-2 virions with PheoA rendered the virions non-infectious in a time- and dose-dependent manner. These observations are similar to those reported by other groups in other infection models [30,32]. The virtual lack of activity of PheoA at post-entry levels may be explained by the fact that PheoA acts primarily on the viral particle, or that PheoA requires longer incubation periods to penetrate the cell and interfere with downstream steps of the virus lifecycle. Given that overall effectiveness of PheoA as virucidal is substantially stronger than during multiple cycle infection experiments, it is likely that virucidal activity is the main mechanism for interference with SARS-CoV-2 infection.

PheoA has been shown to integrate into biological membranes [35]. Thus, it is possible that PheoA could insert into the viral envelope lipid bilayer, altering its biophysical properties, or even disrupting it, thus rendering the virion non-infectious. This would explain PheoA’s virucidal activity and its broad-spectrum among enveloped viruses. PheoA is a plant derived porphyrin closely related to animal porphyrins, which have been widely described as broad-spectrum virucidals (reviewed in Sh. Lebedeva et al. [36]). Virion inactivation is thought to occur through incorporation of porphyrins into the viral envelope membrane and modifying its physico-chemical properties, thus interfering with host cell recognition and fusion processes. However, porphyrins such as protoporphyrin IX display antiviral activity independently of their virucidal activity at post-entry steps and have been proposed to interfere with receptor (ACE2) recognition in SARS-CoV-2 infection models [6]. The structural resemblance between PheoA and proporphyrin IX may explain their similar antiviral properties (broad spectrum and virucidal), but, at the same time, their differences may contribute to PheoA’s increased tolerability and in vivo effectiveness, an issue that has extensively been explored for different PheoA applications as photosensitizer in photodynamic therapies against cancer [31,37].

One huge advantage of PheoA is that it is readily available from plant and algae chlorophyll. PheoA is the dephytylation and demetallation product of chlorophyll *a*, processes mediated by chlorophyllase and Mg-dechelatase, respectively [38,39]. Clorophyllase activity is favored by high temperatures (60–80 °C) [38] and its accumulation varies throughout plant development and in stress conditions. In this study, we also exploited stress conditions (heat) that favour PheoA accumulation and PheoA was semisynthetically prepared from *M. polymorpha* in good overall yield.

Another advantage of PheoA is that its combination with RMDV has an additive effect with no cross inhibition in their antiviral activity, and a mild synergy. This, together with its low toxicity in vivo, represents an advantage that could be clinically exploited [40].

## 4. Materials and Methods

### 4.1. Equipment and Reagents

All solvents were of ACS quality unless stated otherwise. Commercially available PheoA was purchased from Santacruz Biotechnology (>90% by HPLC). A Geno Grinder Spex/SamplePrep 2010 was employed for tissue homogenization. Glass- or aluminum-supported Silica gel 60 (Merck) was used for preparative and analytical TLCs, respectively; for flash column purification, silica gel 60 Å, 230–400 mesh, 40–63 µm was employed. HPLC-UV-MS analysis was carried out by using a Waters Separations module Alliance e2695 system, a Waters QDa Detector Acquity QDa and a Waters Photodiode Array Detector 2996. HPLC was performed by using HPLC grade solvents and a Sunfire C18 (4.6 × 50 mm, 3.5 μm) column at 30 °C, with a flow rate of 1 mL/min and a mobile phase gradient from 70 to 95 of A (formic acid 0.1% in CH_3_CN) in B (0.1% of formic acid in H_2_O) for 10 min. Electrospray in positive mode was used for ionization. The HR-MS analysis was carried out by using an Agilent 1200 Series LC system (equipped with a binary pump, an autosampler, and a column oven) coupled to a 6520 quadrupole-time of flight (QTOF) mass spectrometer. CH_3_CN:H_2_O (75:25, *v*/*v*) was used as the mobile phase at 0.2 mL/min. The ionization source was an ESI interface working in the positive-ion mode. The electrospray voltage was set at 4.5 kV, the fragmentor voltage at 150 V and the drying gas temperature at 300 °C. Nitrogen (99.5% purity) was used as nebulizer (207 kPa) and drying gas (6 L/min). The scan range was 50–1100 *m*/*z*.

### 4.2. Preparation of Crude M. polymorpha Extracts

Plant material (10 g, fresh weight) was collected from each genotype (WT, Mpcoi1–2 and Mpc1hdz) and oven dried (60 °C) until constant weight. The dry tissue was ground to a fine powder using a Geno/Grinder (2 × 2 min at 2700 rpm) and extracted two times at room temperature with 30 mL of CHCl_3_:MeOH (2:1, *v*/*v*) for at least 6 h each time. Extracts were combined and concentrated under a nitrogen flow to produce a dark green residue. The remaining solid was dissolved in DMSO to create stock solutions of ca. 90 mg/mL, which were later employed in the bioassays.

### 4.3. Chromatographic Fractionation of Extracts

Plants extracts were prepared as described above starting from ca. 20 g of plant material and directly subjected to silica gel flash column chromatography employing a solvent polarity gradient starting at *n*-hexane (100%) up to AcOEt:MeOH (4:1, *v*/*v*). A total of 56 metabolite-enriched fractions were obtained. Fractions were analyzed by TLC and those of similar composition were combined to render 12 new pooled fractions, which were screened for antiviral activity.

### 4.4. Preparative TLCs

Photosynthetic metabolites were extracted [two times, o/n, acetone (30 mL)] from fresh, finely grounded *M. polymorpha* thallus (ca. 40 g). The combined extracts were concentrated to a final volume of 10 mL, centrifuged (4000 rcf), filtrated (45 μm, Whatmann PTFE filters) and chromatographed on preparative TLC plates employing the solvent system AcOEt:MeOH (9:1, *v*/*v*). Selected fluorescent spots (C, D and E) were scraped off, eluted (AcOEt:MeOH, 4:1, *v*/*v*) and dried under a nitrogen stream. Single components (C, D and E) were prepared at a 10 mg/mL and submitted to both antiviral assays, as described below, and HPLC-UV-MS analysis as described above.

### 4.5. Semisynthetic Preparation of PheoA

Fresh *M. polymorpha* thallus (ca. 50 g) was oven dried to produce ca. 3.5 g of dry material, which was ground to a fine powder in the GinoGrinder as described above. The obtained powder (3 g) was extracted (three times, Acetone, 90 mL), the extracts combined and silica gel (6 g, ratio 2:1 by weight relative to the dried leaf powder) added. The solvent was evaporated under reduced pressure to produce impregnated silica, which was heated (60 °C) overnight to further potentiate PheoA production. The obtained silica was directly loaded into a flash column that was run as follows: column ID = 3 cm, silica (70 g), *n*-hexane: AcOEt (1:1, 300 mL), AcOEt: MeOH (9:1, 300 mL; 4:1, 600 mL; 7:3, 300 mL), fractions of ca. 35 mL were collected. Fractions were analyzed by TLC and those containing PheoA were pooled to produce PheoA (3.9 mg, 0.13% from oven dried leaf material).

Commercial PheoA was purchased from Santa Cruz and dissolved in DMSO at a concentration of 1 mg/mL. Dilutions were prepared directly in the cell culture media at the concentrations described in the figures.

### 4.6. Cell Culture

Vero E6 (ATCC) and Calu3 (ATCC) cell lines were kindly provided by Dr. Enjuanes (CNB-CSIC). A549 cells were kindly provided by Dr. Juan Ortín (CNB-CSIC) and Huh7 cells were kindly provided by Dr. Chisari (TSRI, La Jolla). A549 and Huh7 cells were transduced with a retroviral vector enabling expression of ACE2 in a di-cistronic expression cassette also conferring resistance to blasticidine. Transduced populations were selected using 2.5 µg/mL of blasticidine. All cell cultures were kept in complete media (DMEM) supplemented with 10 mM HEPES, 1X non-essential amino acids (GIBCO), 100 U/mL penicillin-streptomycin (GIBCO) and 10% fetal bovine serum (FBS; heat-inactivated at 56 °C for 30 min). Unless otherwise stated, all infection experiments were performed at 37 °C in a CO_2_ incubator (5% CO_2_) the presence of 2% FBS and in the absence of selection antibiotics.

### 4.7. Viruses

SARS-CoV-2 (*Coronaviridae; Orthocoronavirinae*; *Betacoronavirus*; *Sarbecovirus*; strain NL/2020) was kindly provided by Dr. R. Molenkamp, Erasmus University Medical Center Rotterdam. SARS-CoV-2 stocks were produced and titrated in VeroE6 cells by inoculation at a multiplicity of infection (MOI) of 0.001 TCID_50_/cell. Cell supernatants were harvested at 72 hpi, cleared by centrifugation, aliquoted, and stored at −80 °C. SARS-CoV-2 virus titers were determined by endpoint dilution and cytopathic effect determination using the tissue culture infective dose fifty (TCID_50_) method by (Reed and Muench, 1938) [41] and expressing the infectivity titers as TCID_50_/mL. Additionally, infectivity titers were estimated using endpoint dilutions and immunofluorescence microscopy, as described below. This approach enables determining the number of infectious focus formation unit (FFU) and expressing infectivity as FFU/mL. Both readouts produced comparable SARS-CoV-2 infectivity titers and were used to determine optimal virus dose (multiplicity of infection [MOI]; infectious units/cell) for each experiment.

VSV-GFP [42] was kindly provided by Dr. Rodriguez (CNB-CSIC). WNV-GFP recombinant virus was rescued from cloned cDNA as reported previously [43]. Trans-complemented defective reporter HCV virions (HCVtcp) were produced and used as described in Steinmann et al. [44]. The hCoV-229E-GFP [45] was kindly provided by Dr. Thiel (University of Basel) and propagated in Huh7 cells at 33 °C in a controlled 5% CO_2_ environment. GFP-expressing recombinant virus (WNV-GFP, 229E-GFP or VSV-GFP) infectivity was also determined as FFU/mL using endpoint dilution and fluorescence microscopy.

### 4.8. Cypopathic Effect Protection Assays in Vero E6 and Huh7-ACE2 Cells

Vero E6 or Huh7-ACE2 cell monolayers were inoculated (MOI = 0.001) in the presence of a wide range of two-fold dilutions of the crude, or partially purified extracts, or pure compounds and incubated for 72 h. Cytopathic effect and lack thereof was visualized by crystal violet staining, as previously described [17]. Untreated and solvent-treated cells were included in each plate as controls. Assayed samples were pronounced protective when three or more consecutive two-fold serial dilutions (regardless of their final concentration) fully prevented SARS-CoV2-induced antiviral activity as determined by comparison with infected and uninfected control well staining in each experimental plate. A minimum virus dose (10 infectious units/well; MOI 0.001 in 1e4 cells/well) capable of producing overt cytopathic effect was used. Lower virus doses produce visible plaques and complete absence of CPE coincides with the expected endpoint dilution, around 8-fold lower dilutions.

### 4.9. Evaluation of the Antiviral Activity by Immunofluorescence Microscopy

Vero E6, A549-ACE2 or Calu3 were seeded onto 96-well plates as described above and infected in the presence of the indicated compound dose (MOI = 0.01). Twenty-four hours post infection (48 h for Calu3 cells), cells were fixed for 20 min at room temperature with a 4% formaldehyde solution in PBS, washed twice with PBS and incubated with incubation buffer (3% BSA; 0.3% Triton X100 in PBS) for 1 h. A monoclonal antibody against the N protein was diluted in the incubation buffer (1:2000, *v*/*v*; Genetex HL344) and incubated with the cells for 1 h; after this time, cells were washed with PBS and subsequently incubated with a 1:500 (*v*/*v*) dilution of a goat anti-rabbit conjugated to Alexa 488 (Invitrogen-Carlsbad, Carlsbad, CA, USA). Nuclei were stained with DAPI (Life Technologies, Budapest, Hungary) as recommended by the manufacturer during the secondary antibody incubation. Cells were washed with PBS and imaged using an automated multimode reader Spark Cyto (TECAN-Grödig, Austria).

All the infection experiments were performed by mixing the virus and compound dilutions 1:1 (*v*/*v*) before addition to the target cells. In the time-of-addition experiments, Vero E6 cultures were inoculated (MOI from 0.5–1) for 1 h in the presence or absence of the compounds at 37 °C. Subsequently, virus-compound mixtures were left at all times, or removed and replaced with fresh 2% FBS complete media containing or not the tested compounds (see experimental scheme in Figure 7 for details). Cells were fixed 6 h post-inoculation.

### 4.10. Viral RNA Quantitation by RT-qPCR

A549-ACE2 cell monolayers were inoculated at MOI = 0.001 in the presence of non-toxic concentrations of the compound. Forty-eight hours later, cell supernatants were collected and heat-inactivated as described in (Smyrlaki et al., 2020) [46], and processed directly for RT-qPCR. Alternatively, cell lysates were prepared using the Trizol reagent (Thermo Scientific) and the viral RNA content was determined by RT-qPCR using previously validated sets of primers and probes specific for the detection of the SARS-CoV-2 E gene and the cellular 18S gene, for normalization purposes. ∆Ct method was used for relative quantitation of the intracellular viral RNA accumulation in compound-treated cells compared to the levels in infected cells treated with DMSO (set as 100%). Values obtained in mock-infected cells were used to determine the assay limit of detection (LOD).

### 4.11. Cytotoxicity Measurement by MTT Assays

Cell monolayers were seeded in 96-well plates. The day after cells were treated with a wide range of compound concentrations and forty-eight hours later they were subjected to an MTT assays using standard procedures [47]. The CC_50_ values were graphically interpolated from dose–response curves obtained with three biological replicates.

### 4.12. Assessment of Viral Entry Using Retroviral Pseudotypes

Retroviral particles pseudotyped with SARS-2-CoV spike envelope protein (SARS2pp) were produced in HEK293T cells as previously described [34] with materials kindly provided by Dr. F. L. Cosset (INSERM, Lyon) and J. M. Casasnovas and J. G. Arriaza (CNB-CSIC) for the S protein cDNA. Particles devoid of envelope glycoproteins were produced in parallel as controls.

### 4.13. Statitistical Analysis

Descriptive statistics were calculated using Microsoft Excel. One-way ANOVA and post hoc tests were calculated using IBM SPSS Software Package (version 26). EC_50_ and EC_90_ values were obtained employing the PROBIT regression method [48] using IBM SPSS vs26, except for values in Table 1 that were calculated by graphic interpolation of individual curves.

Synergy analysis was carried out in the web-based platform Synergy Finder (https://synergyfinder.fimm.fi/, accessed on 25 September 2021) [29].

## 5. Conclusions

The chlorophyll derivative PheoA, a porphyrin compound similar to the animal Protoporphyrin IX, has an extraordinary antiviral activity against SARS-CoV-2 preventing infection of cultured monkey and human cells, without noticeable cytotoxicity.

PheoA interferes with viral infectivity in a dose- and time-dependent manner.

Besides SARS-CoV-2, PheoA displayed a broad-spectrum antiviral activity against (+) strand RNA viral pathogens such as HCV, West Nile, and other coronaviruses.

Our results indicate that PheoA displays a remarkable potency and a satisfactory therapeutic index, which together with its previous use in photoactivable cancer therapy in humans, suggest that it may be considered as a potential candidate for antiviral therapy against SARS-CoV-2.

## Figures and Tables

**Table 1:** Potency and cytotoxicity indexes of commercially available PheoA. Values are provided as mean and standard deviation of three biological replicates (*n* = 3).

Cell Line	EC_50_ (nM)	EC_90_ (nM)	CC_50_ (nM)
Vero-E6 (Green Monkey)	54 ± 13	106 ± 16	>8420
A549-ACE2 (Human Lung)	62 ± 25	201 ± 84	2600 ± 500
Calu3 (Human Lung)	35 ± 20	98 ± 8	>8420

## Data Availability

Data is contained within the article.

## Supplementary Materials

The following are available online at https://www.mdpi.com/article/10.3390/ph14101048/s1, Figure S1: RMDV interferes with SARS-CoV-2-induced cytopathic effect, Figure S2: Crude extracts of several plant species show antiviral potential against SARS-CoV-2 infection in cell culture, Figure S3: TLCs of pooled fractions submitted to antiviral assays, Figure S4: Scheme of PheoA preparation from plant material. KG, Silica gel 60, Figure S5: PheoA protects Vero-E6 and human hepatoma Huh7 cells from virus-induced cytopathic effect, Figure S6: Semi-synthetic PheoA preparations display antiviral activity against SARS-CoV-2, Figure S7: Pyropheophorbide a displays antiviral activity against SARS-CoV-2, Figure S8: SARS-CoV-2-pseudotyped retroviral vectors (SARS2pp) are not susceptible to PheoA antiviral activity.
